# Standardising and Assessing Digital Images for Use in Clinical Trials: A Practical, Reproducible Method That Blinds the Assessor to Treatment Allocation

**DOI:** 10.1371/journal.pone.0110395

**Published:** 2014-11-06

**Authors:** Asha C. Bowen, Kara Burns, Steven Y. C. Tong, Ross M. Andrews, Robyn Liddle, Irene M. O′Meara, Darren W. Westphal, Jonathan R. Carapetis

**Affiliations:** 1 Menzies School of Health Research, Charles Darwin University, Darwin, NT, Australia; 2 Royal Darwin Hospital, Darwin, NT, Australia; 3 School of Business, Queensland University of Technology, Brisbane, QLD, Australia; 4 Telethon Kids Institute for Child Health Research, University of Western Australia, Perth, WA, Australia; 5 Princess Margaret Hospital for Children, Perth, WA, Australia; The University of Queensland, Australia

## Abstract

With the increasing availability of high quality digital cameras that are easily operated by the non-professional photographer, the utility of using digital images to assess endpoints in clinical research of skin lesions has growing acceptance. However, rigorous protocols and description of experiences for digital image collection and assessment are not readily available, particularly for research conducted in remote settings. We describe the development and evaluation of a protocol for digital image collection by the non-professional photographer in a remote setting research trial, together with a novel methodology for assessment of clinical outcomes by an expert panel blinded to treatment allocation.

## Introduction

Telemedicine is increasing in popularity, particularly for dermatologists and other specialities where a clinician is not always onsite for direct patient care [1]. With the increasing availability of cheap, simple, high quality digital cameras for the non-professional photographer, often a clinician or auxiliary [2], [3], the appeal of utilising digital images to diagnose or monitor treatment progress is growing. An extension of this application is the use of digital images of cutaneous disease to assess outcomes for intervention studies including randomised controlled trials (RCTs) [4]. Advantages include: maintaining blinding of the outcome assessor to treatment allocation; scoring digital images in batches to improve work flow; and utilising expert outcome assessors remote from the site of data collection. These advantages have particular appeal for the conduct of research in remote regions or for multicentre studies where standardisation of clinical outcome measures is critical. However, published methodologies to guide such use of digital images are lacking. In addition, unlike hospital or clinic based health photography which is usually performed in a dedicated setting with instruments and lighting operated by a professional photographer [5], [6], this ideal may not be achievable in remote, field-based research. Therefore we aimed to develop a method to facilitate the acquisition of consistent, high quality digital images of superficial skin lesions in the context of conducting a RCT in a remote setting. A medical photographer (KB) guided this process [4], [7]. The digital images were taken by field research staff and allowed the assessment of outcomes in a blinded manner.

The four characteristics of an excellent clinical photograph are correct perspective, use of a scale aligned with the image frame, even lighting and a neutral background [8]. To achieve these characteristics, it is important to standardise the equipment, camera settings, participant positioning and photography technique so that reproducible images are captured [9], [10]. Once images of satisfactory quality have been captured, consideration must be given to storing these confidential images for future use. Standardised protocols that address these priorities for collecting digital images are not available in the peer-reviewed literature.

Impetigo trials are an example of cutaneous disease research where digital images can improve the objectivity of outcome assessment. Impetigo is a common, non-benign cutaneous infection that mostly occurs in resource-limited contexts [11], affecting >2% of the global population at any one time [12]. Impetigo also regularly affects school-aged children in industrialised settings [13]. Treatment of impetigo is a public health priority to prevent severe sequelae including streptococcal and staphylococcal sepsis, focal invasive disease, post-streptococcal glomerulonephritis (which is in turn linked with chronic renal failure) [14], and a postulated causative link with rheumatic fever and rheumatic heart disease [15], [16]. These sequelae mainly occur in resource-limited settings and are responsible for hundreds of thousands of deaths each year globally [11].

The authors of a meta-analysis on interventions for impetigo recommended the use of clear and objective outcome measures for future impetigo research [17]. As the burden of impetigo is in resource-limited contexts where ready access to clinicians for immediate end-point assessment may not be feasible, digital image end-points are appealing and objective. The only RCT from a resource-limited context included in the meta-analysis reported the use of photographs for outcome assessment. In this RCT, successful treatment was defined as clinical cure or marked improvement with an additional measure using photographs, but they did not report the methodology of image collection or how they determined the outcome based on this end-point [18]. Well-defined, reproducible endpoints are needed and blinded outcomes are the cornerstone of good clinical trial design [19].

In conducting a RCT on impetigo treatment in a remote setting, we developed a standardised, reproducible method for collecting images of skin sores at different time points [6]. Reviewers blinded to treatment allocation assessed the images using a simple, reproducible, quick method that provided readily analysable data. The image capture protocol and novel methodology for blinded assessment may be useful for other trials in cutaneous disease research.

## Method

### Ethics

This study and all consent documentation were approved by the Human Research Ethics Committee of the Northern Territory Department of Health and Menzies School of Health Research (HREC 09/08). Indigenous elders provided community consent before recruitment commenced. The parent or legal guardian for all participants provided written informed consent. The study was explained by a local interpreter or by using a talking book in the participant's first language. Written consent was itemised for all study procedures including the collection of digital images.

### Setting

We conducted a non-inferiority RCT in 7 remote Indigenous communities of the Northern Territory of Australia between 2009 and 2012 [20]. The research team was based in Darwin and travelled via plane or road to the remote communities up to 1500 kilometres away. There were 663 episodes of impetigo (in 508 children) enrolled in the trial and each had either one or two sores under investigation. Research assistants trained in the photography protocol captured digital images of all trial participants' sores on days 0, 2 and 7. Images were stored electronically. A panel of paediatricians specialising in the care of Indigenous Australian children externally reviewed these digital images at a later date, according to a standardised scoring system as reported below.

### Details of the protocol for capturing digital images of impetigo

#### Equipment

**Figure 1:**
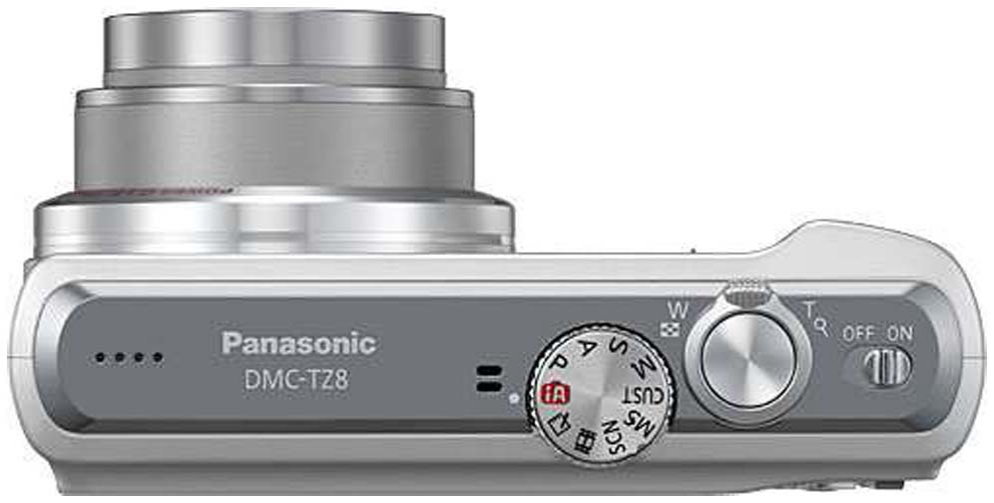
The Panasonic DMC-TZ8 camera demonstrating zoom and camera settings as indicated in figure 5. http://www.digitalcamerawarehouse.com.au/prod6699.htm, last accessed 28 September 2014).

**Figure 2:**
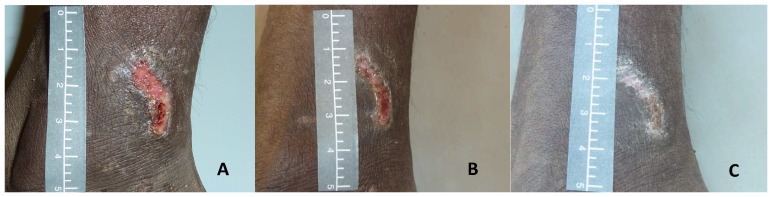
Use of scale to define the upper and lower boundaries of image in the landscape position. This series of images of the same sore on days 0 (A), 2 (B) and 7 (C) utilise the scale well with the 0 at the top of the image and the 5 at the bottom, are clear and focussed and demonstrate sore healing over time. Limitations include different availability of light as captured during different parts of the day using outdoor light.

**Figure 3:**
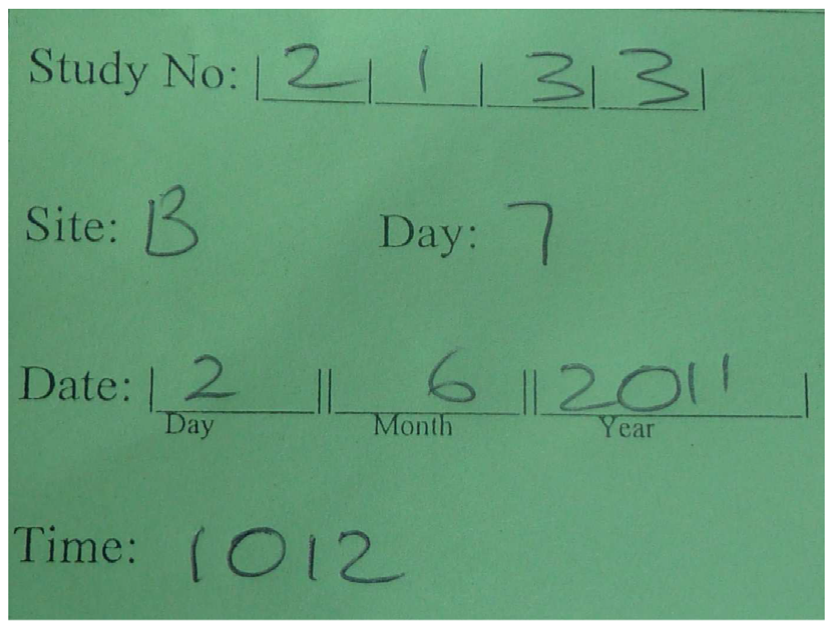
An example of the participant identification card described in table 1. This card contains participant number, date of image, study day, and whether it is sore A or B as up to two-thirds of study participants had two sores enrolled in the study.

**Figure 4:**
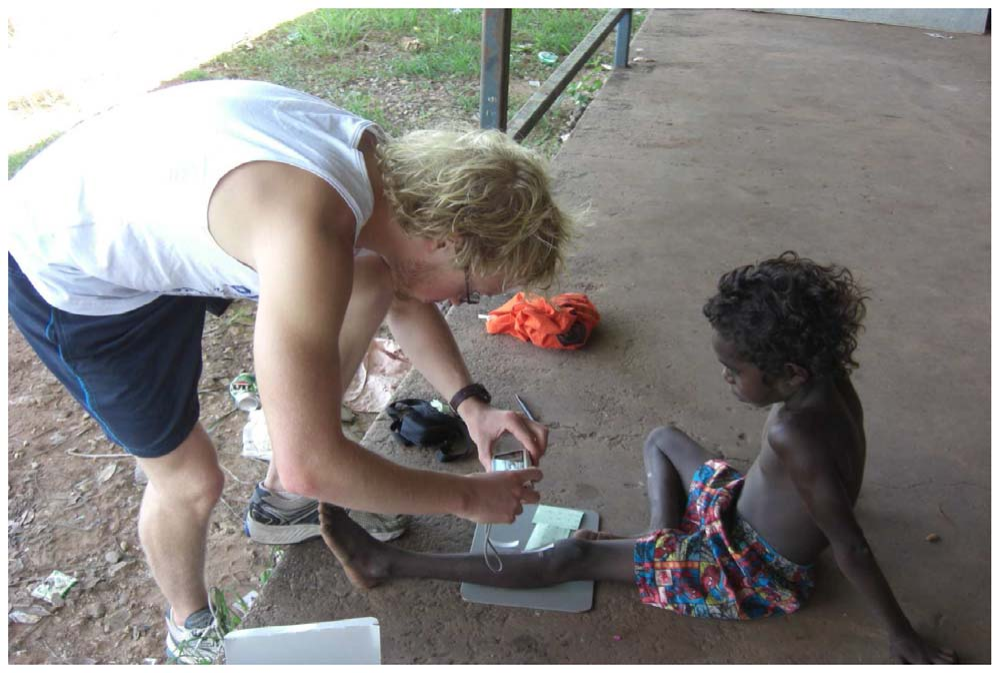
Capturing the image using the study camera, grey background, and grey scale in a remote context. The individuals in this image have given written informed consent to publish this image.

All images were captured using standardised equipment (table 1, figures 1–4).

**Table 1:** Study equipment chosen including the required features, advantages and alternatives available or recommended in the literature.

Item	Specific Choice	Features	Advantages	Alternatives
**Digital Camera**	Panasonic LUMIX DMC-TZ8 14.5 megapixel digital camera (figure 1)	Macro to 3 cm from a 12X zoom lens equivalent to a 25–300 mm lens on an SLR	Inexpensive	Digital single lens reflex (SLR) camera [32]
		Built in flash	Readily available	
		Rugged metal casing	Automatic	
		Lithium ion rechargeable battery	Pre-specified settings to achieve uniform, reproducible images whilst using amateur photographers	
		4GB memory card		
**Background**	Grey background (figure 4)	A small grey board positioned behind the body part being assessed	Transportable and light weight	Green or grey surgical drapes [33]
			Manoeuvrable	
			Non-reflective	
			Neutral	
**Scale** [34]–[36]	5cm, non-reflective grey scale (figure 2)	Vertical scale	Defined the upper and lower boundaries of the photograph (figure 2)	Paper or commercially available scale e.g. the ABFO scale [34]
		Single use sticker	Easily removed and discarded	
			Good infection control	
**Identification Card**	Cardboard handwritten card (figure 3)	Pre-formatted to add individual randomisation number, study visit day, sore number, date and time of image capture	Maintained study blinding	
			Photographed prior to impetigo image capture to avoid inclusion of any identifying details in the image for scoring by blinded reviewers[37]	
			When forgotten, identification card was photographed upside down immediately after the series of sores was captured [37]	

#### Camera Settings

**Figure 5:**
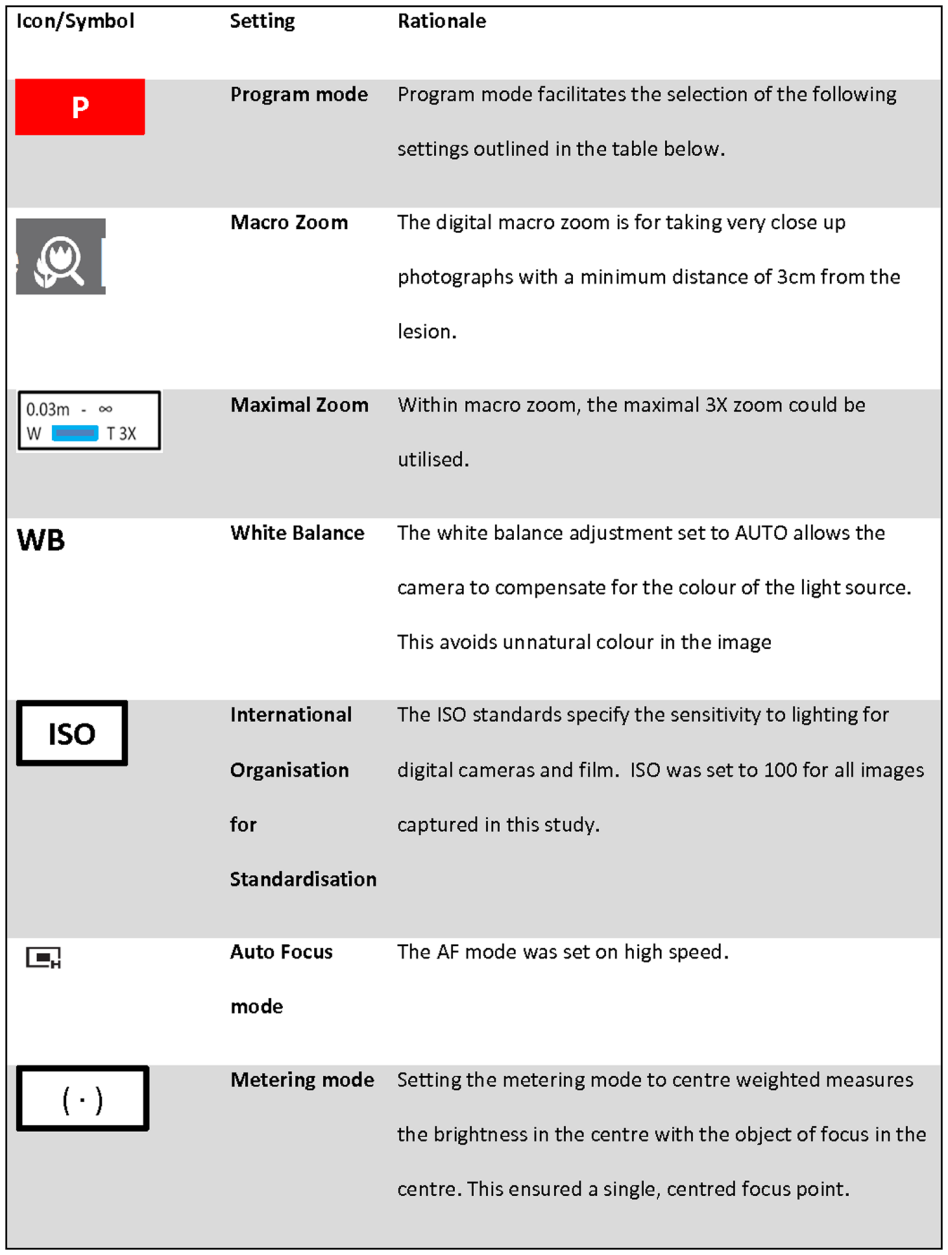
The camera settings and icons as used in the protocol (these icons and settings are standard across other popular camera models). In addition the rationale is provided on why these settings were adopted.

Due to data collection occurring simultaneously in more than one community, we purchased three identical cameras. All study camera settings were programmed by the study manager (figure 5) and checked against the standard operating procedure (SOP) by the research assistants before each use. Training in the programming of settings and operation of the camera was conducted with each new research assistant and image quality reviewed at the completion of most field work trips. A quick list to summarise the process for image capture in the field was developed (table 2).

**Table 2:** Quick List for capturing standardised photographs.

Step	Action
**1**	Confirm all camera settings are correct (figure 5), participant, paperwork and previous image for orientation (if available)
**2**	Position participant in the shade: comfort, lesion exposed, neutral background, scale in the same plane as the lesion
**3**	Photograph participant ID (table 1) prior to capturing series to preserve blinding.
**4**	Position camera in same plane as sore (figure 6). Centre the sore and focus camera. Take minimum of 3 photos. Take additional photographs if none are clear and focussed.
**5**	Record photograph number and notes in participant's file
**6**	Save digital images in secure location and delete from camera

This could be printed on a small card to be carried with the camera as a reminder to research assistants capturing images.

#### Lighting Conditions

The main light source was the flash as force flash was always on. This provided a standard light source for all three cameras in all settings. In addition, preference was given to capturing digital images outdoors in the shade to improve the ability of the camera to focus on the subject and avoid distracting shadows [10]. Direct sunlight was avoided to minimise the potential of a stronger light source resulting in an over-exposed image. For uniformity of conditions, when it was not possible to photograph participants outdoors, maximal ambient light was achieved by turning on all lights and opening any curtains or doors. Night time photography was avoided by returning to photograph the participant first thing the following day.

#### Positioning of study participant

**Figure 6:**
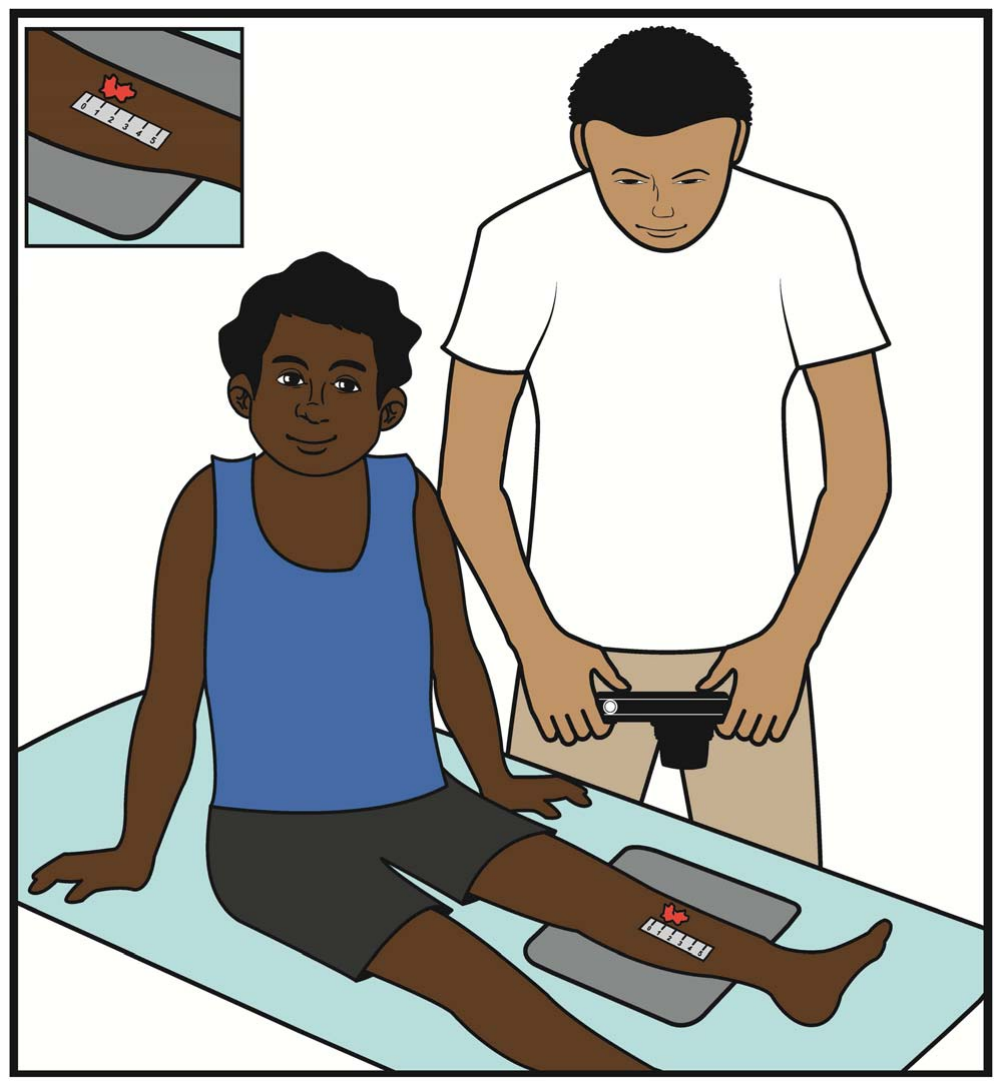
Cartoon demonstrating the photographer, camera and sore were in the same plane to optimise reproducibility of captured images

As we were working with children, prior to taking any photographs, the participants were reminded to remain still and the carer was engaged in reassuring the child. The participant was positioned comfortably in a chair or on the floor with a neutral grey background beneath the limb or site to be photographed (figures 4 and 6). Jewellery, clothing or hair that might obscure the area of interest were removed or tied back. Any dressings covering the lesion were also removed.

Once settings were rechecked, a 5 cm neutral grey scale was placed in a vertical position, in the same plane as the sore, as close as possible to the left of the sore without obscuring any edges of the lesion (figures 2 and 6). The upper limit (0) of the scale was positioned at the top of the frame and the lower limit (5) at the bottom. This ensured all images were captured at the same scale so that when paired images were reviewed the sores were comparable and any reduction in size could be assessed as a measure of sore healing.

### Photography technique

To maximise sharpness by depth of field, the camera lens plane was positioned parallel to the sore plane with the photographer standing above the sore (figure 6). The sore was centred using the white square corners at the centre of the camera screen. The shutter was depressed halfway to focus the lesion prior to capturing the image.

A minimum of three images of each sore were taken at each time point, to ensure that at least one adequate image was available for outcome assessment, a technique known as bracketing [21]. The research assistant was instructed to check each image for SOP conformity and to take additional photographs if a clear, focussed image showing all details of the sore had not been obtained. Each photograph number was recorded in the respective participant's case report form (CRF).

Once all images had been captured, brief notes were made in the participant's CRF to describe the positioning of the participant so that whenever possible the same position could be used for future images. Further follow up images of the same sore were required on day 2 and day 7 from enrolment. For consistency of orientation, previous images of the same sore were checked on the study camera before capturing the next image.

### Image download and storage

Standardisation of image storage is critical to the meticulous utilisation of this method [9], [22]. At the completion of each day, the image files were downloaded from the camera memory card to a password-protected laptop. The laptop files served as a data backup, which was important given that study visits lasted between two and three weeks and internet was not reliable enough in the remote context to upload numerous large files every day. Upon return of the team to the research centre in Darwin; all images were downloaded from the camera to the main computer server where daily backups occur. All images were taken and stored in high quality.JPEG (Joint Photographic Experts Group) format for convenience as our chosen camera did not shoot in an uncompressed (‘raw’ or ‘ loss-less’) format. There are limitations to using a compressed or ‘lossy’ format but the benefits of using a compact camera and storage of images for comparison outweighed these and is in line with other clinical studies [23]. Three copies of each unmodified image were saved: one in the participant's folder labelled with participant number; one in the generic backup folder labelled with the camera-generated photograph number as recorded in the participant CRF; and one labelled with a randomly generated number between 1 and 15 000. Only once this had occurred were images deleted from the memory card. These re-identifiable images are to be stored on a secure server for up to 25 years, in keeping with ethical requirements for research in children [24]. From the three available digital images of each sore, the best quality image (key criteria were focus, exposure and magnification) was selected for outcome assessments.

### Quality control process

**Figure 7:**
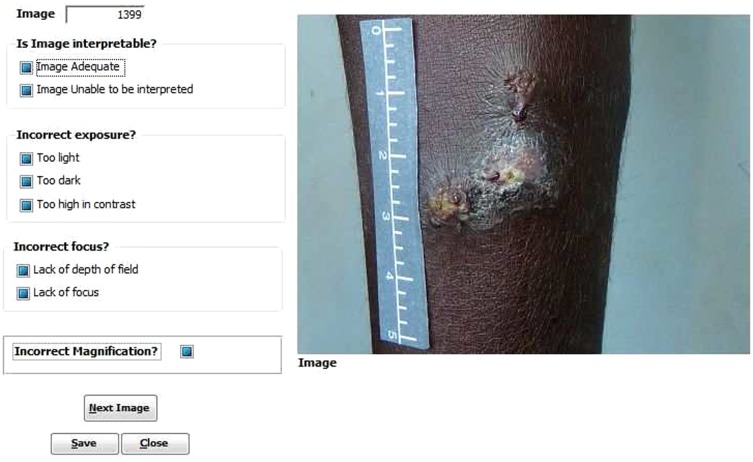
Database form used for Quality Control check by medical photographer.

As all images were collected by amateur photographers, images were regularly checked by the study doctor (AB) and feedback provided if the image did not conform to the SOP. In addition, prior to commencing primary outcome reviews, a quality control (QC) check of all available digital images was performed mid-way through the study (collected from the first 200 study participants) by a medical photographer (KB) experienced in capturing digital images of skin conditions. The QC was a priority in this study as the method described had not been previously used or evaluated and we were unsure whether digital image manipulation might be needed for image scoring. Digital images can be manipulated to overcome flaws in image capture [21], however this has limitations. Our *a priori* hypothesis was that digital image manipulation would not be needed. To confirm this after recruitment of 200 participants, 1 300 images were scored as either adequate or unable to be interpreted using the definitions in table 3. If “unable to be interpreted” was chosen, the reviewer was instructed to provide reasons (figure 7).

**Table 3:** Definitions used for the quality control assessment of digital images.

Assessment	Definition
**Adequate**	The entire sore was seen in enough detail to determine the margin and most of the interior; AND the scale was seen in sufficient detail to determine the approximate size.
**Unable to be interpreted**	The image of the sore could not be interpreted due to incorrect exposure (too light or too dark), focus (lack of focus or depth of field) or incorrect magnification.

### Methods for digital image assessment

We developed a method for scoring digital image pairs that was simple, limited bias, quick and afforded readily analysable data. As the methodology was novel, a paper-based pilot was conducted employing 13 clinicians and researchers to confirm usability prior to building an automated database for scoring. In the pilot, 22 paired digital images from 10 participants of either day 0 and 2 (5 participants) or day 0 and 7 (6 participants) were reviewed in random order (days 0/2 or 2/0 and 0/7 or 7/0) by the 13 reviewers. The initial definition of healing or improved included both a visual description of the sore pair and a clinical decision as to whether further antibiotic treatment was needed.

**Figure 8:**
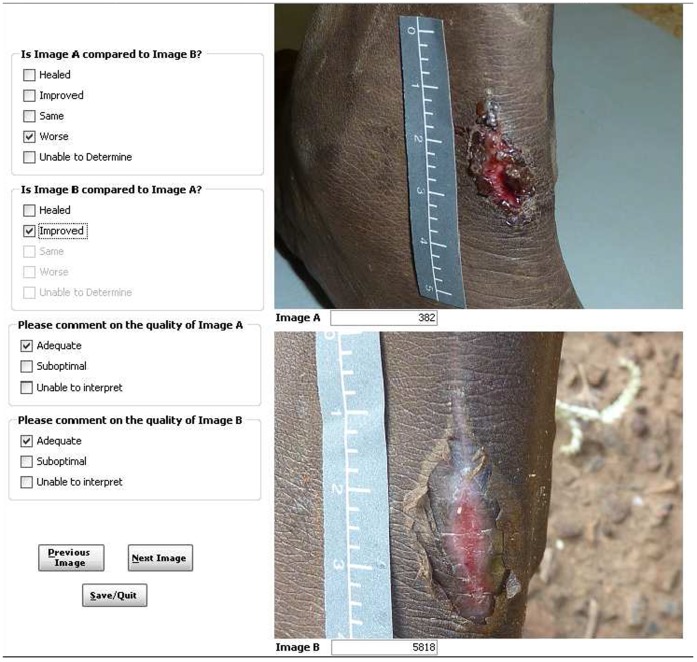
This shows the database format utilised for scoring digital image pairs. Image A was taken on day 0 and image B on day 7. Image B shows erythema and as such was scored as improved using the definitions in table 4.

The primary outcome for the RCT was treatment success at day 7 according to paired digital image scoring. After the successful pilot, the digital image pairs were organised in random order in a purpose built database (figure 8) using the randomly assigned number between 0 and 15 000 as the only identifying information. Scoring of the digital image pairs was on non-standardised computer screens at locations remote from the primary study site. Scoring was by a group of eight paediatricians with expertise in caring for Indigenous children with impetigo. Primary outcome reviewers blinded to treatment allocation were provided pairs of images from day 0 and 7 (or day 0 and 2) in random order. Each reviewer was unaware of which image (A or B) was pre- or post-treatment and was asked to decide if image A, compared to image B, was healed, improved, the same, worse, or unable to be determined using the definitions (table 4) and vice versa (i.e., image B compared to image A). To expedite this process, an auto-fill was used in the database. For example, when image A was scored as “worse”, auto-fill made available the options of “healed” or “improved” only for the comparison of image B to image A. Where “healed” or “improved” were selected for image A, auto-fill completed the scoring with “worse” for image B. The use of auto-fill made the scoring process as rapid as possible. Thus reviewers were blinded to both treatment allocation and the chronological order of sores. Every image pair was evaluated by two independent reviewers from the panel of eight. Where disagreements occurred, an expert panel of three determined the final result by consensus.

**Table 4:** Definitions used for final outcome scoring of digital images of impetigo.

Assessment	Criteria	Outcome
**Healed**	Lesions no longer evident or flat, with no evidence of crusting, erythema or purulence, but possibly with evidence of hyper- or hypo-pigmentation where the original sore was located.	Success
**Improved**	Lesions reduced in sore diameter and erythema; AND progression from blister to crusting and flattening of the sore. Purulence not evident.	
**Same**	No appreciable change in diameter, erythema or purulence of lesion.	Failure
**Worse**	Increase in diameter, erythema or purulence of lesion.	
**Unable to be determined**		

Following un-blinding of the chronological order of sores and to produce readily analysable results from the blinded scoring system, if image B was the day 2 or 7 sore, treatment success was deemed to have occurred if image B was healed or improved compared to image A (day 0 sore). If image B was the day 0 sore, then success was deemed to have occurred if image B was worse compared to image A (day 2 or 7 sores) *and* image A was either healed or improved (table 4).

## Results

### Overall image collection

Over the 3-year study, almost 10 000 digital images were collected and stored by more than 20 research assistants who collected data in 7 remote communities covering an area of 1.35 million km^2^. From all of the images collected, the best available image of the bracketed set was selected for outcome assessment. Approximately 3 300 digital images were required to determine the primary and secondary outcomes of the RCT by analysing the results of paired comparisons. The project manager (IO) reviewed all images and selected the best available image for the paired comparison. The best available image (determined by focus, exposure and magnification) was most often the second or third image captured. This was consistent by study visit day.

Of the 9 944 digital images collected, only 17 (0.17%) were not available for assessment due to staff error. These errors were the wrong site photographed on subsequent days to the original site (n = 6), the photograph not saved or filed (n = 4) and the photograph not taken due to staff error (n = 7).

### Quality control check

For the QC check, 1 300 digital images from the first 200 participants that conformed to the described methodology were reviewed. 1 258 (96.8%) were deemed adequate using the definitions provided. Of the 42 images (3.2%) deemed unable to be interpreted there was some overlap in categorisation: 29 were due to incorrect exposure (8 too light, 21 too dark), 16 were due to lack of focus, 2 had incorrect magnification due to lack of focus and 1 had incorrect magnification (Table S1). Results of the QC review were reassuring and consequently digital image modification was not required.

### Pilot for digital image scoring

Thirteen reviewers piloted the digital image scoring process. All pilot reviewers agreed the process was quick and manageable with image quality being adequate. Initially definitions for healing, improved, same or worse included a 2-armed definition with a description of both sore healing and a clinical judgement as to whether treatment with further antibiotics was indicated. The reviewers reported that the need for additional treatment was difficult based on images alone and as the decision had no timely clinical impact, we removed this decision from the definitions.

### Digital image scoring results

Outcome scorers reported 98.3% of digital images as able to be interpreted using the quality codes shown in figure 8. Of these, 89.9% were adequate and 8.4% suboptimal but still able to be interpreted (Table S2). The inter-rater reliability of digital image scoring was moderate. When assessing for treatment success (pooled healed and improved, table 4) versus treatment failure (unchanged or worse), there was 86% agreement between reviewers with a kappa score of 0.4. When assessing using the 5 available definitions (figure 8, table 4) there was 64% agreement between reviewers, with a kappa score of 0.3 (Table S3).

## Discussion

This is the first description of a method for capturing and scoring comparative digital images of skin lesions in clinical research. The methods outlined were practical even in remote contexts, robust, reproducible and simple enough for non-professional photographers to consistently follow. Strengths of the described methodology include the quality control check and more than 98% of captured images being interpretable. The gold standard for needing to retake orthodontic photographs for poor quality was set at 90% [3] and our findings of adequacy were at this level, but when ‘suboptimal but still able to be interpreted' was included exceeded this gold standard. In addition, the described method was followed by more than 20 study staff in remote contexts resulting in <0.2% of images being unavailable for assessment. Digital images were the only available form of documentary evidence for this blinded, clinical trial so it was essential to have a robust process. Our results support that the process outlined works.

The adoption of a standard set of image settings (figure 5) and a Quick List that guided training in the methodology (table 2) facilitated a uniform set of images that did not require any digital manipulation. Guidelines on the manipulation of digital images specify that while “it is acceptable practice to adjust the overall brightness and contrast of a whole image” [25] it is best practice if a group of images are to be compared to each other, that the processing of individual images should be identical. The question of “what constitutes a ‘‘reasonable’’ adjustment of image settings such as brightness and contrast, etc.” has become important for publication in scientific journals and is now included in instructions to authors [26]. For example, the instructions to authors in the Journal of Cell Biology outline that, if manipulation of a digital image is undertaken, these manipulations must not obscure, eliminate, or misrepresent any information present in the original [25]. Forensic guidelines also emphasise this rigorous approach for reproducibility [27]. As the method for capturing digital images reported above had not been previously validated and the outcome was based on a comparison of image pairs, the QC check by a professional medical photographer was an important step in determining whether our images would meet this industry standard. Based on these results, we did not permit the use of photo-editing software to modify any of the images [27]. We recommend following a protocol such as ours that has been subjected to rigorous QC checks for future skin disease research which should largely obviate the need for any digital image manipulation.

High staff turnover when working in remote settings [28] resulted in frequent training and re-training sessions in capturing digital images using the methods described. Educational PowerPoint slides were developed for this purpose and supplemented by the quick reference guides developed (tables 1 and 2, figures 5 and 6). In addition, real time review of captured digital images with feedback to the research assistants was useful. Despite the use of a standard protocol and ongoing training, occasional human errors did occur as described above.

A limitation when using digital images for endpoint assessment is the inability of reviewers to make a clinical decision using the additional senses of hearing (patient feedback on pain and pruritus), touch (warmth, fluctuance) and smell, when provided only with the image. For cutaneous diseases where the appearance of a lesion is the primary determinant of outcome, this limitation can be partly addressed with a robust protocol for capturing reproducible, diagnostic images for outcome assessment. This known limitation impacted upon the inter-rater reliability agreement as physicians were asked as reviewers to use a novel diagnostic modality to score outcomes. To overcome this, we used a consensus panel of three to adjudicate any discrepant scoring. The consensus panel discussed all image pairs until consensus was reached. The possibility that the use of the project manager to select the best available image introduced bias is a possible limitation. However, the QC check by a professional photographer confirms that the perceived bias was minimal with high quality images consistently being provided to reviewers.

We suggest that this protocol could also be adapted from the research setting for use in clinical care. In settings where specialised clinicians are not readily available, standardised digital photography of cutaneous lesions could be used in telemedicine to allow highly skilled clinicians to assist local health staff to manage patients in remote locations.

A unique feature of this protocol for standardising the comparison between image pairs of the same sore where the only detectable difference was changes in the appearance of the lesion [6], was the requirement for all research assistants to check the orientation of the image on the study camera before capturing the next image. Guidelines on doing this were provided. Previous expert advice has been for image capture to be performed consistently by the same photographer [21]. This was not possible within the remote research environment and overall <5% of participants had all 3 days of images collected by the same person. Nonetheless, >99% of image pairs were assessable for the primary outcome. This finding adds to the photography literature. Providing amateur photographers with simple instructions and guidance for collecting digital images using standardised camera settings results in digital images that are of a high quality and can be assessed by blinded, independent reviewers for outcome determination.

A non-inferiority RCT comparing two treatments of a common condition such as impetigo requires rigorous, blinded, objective endpoints for assessment. Here, we have described the method developed based on the available evidence and expertise, for capturing digital images. We report these here for use in subsequent research trials. This method was simple, reproducible and from the QC check provided 97% of images that were adequate for assessment. Whilst other RCTs have used a digital image of the skin as a primary outcome, such as in pyoderma gangrenosum [29], wound healing [30], or pressure sores [31], this is the first report of a standardised, reproducible protocol that has been subjected to a rigorous QC assessment for an impetigo RCT. Our results confirm the reproducibility of the simple resources developed and published herein which will further enhance the rigour of trials using a photographic end-point.

## Conclusions

This is the first report of a standardised, reproducible protocol that has been subjected to a rigorous QC assessment for research involving impetigo and could be adapted for other skin disease research. Our study confirms non-professional photographers are able to capture high quality digital images of skin for this purpose. We present a simple method for capturing high quality digital images of skin sores in a RCT and the methods used to score digital image pairs. Future trials for management of skin conditions, particularly in remote contexts, may benefit from adopting this protocol.

## Supporting Information

Table S1 **Results of quality control (QC) check.** When the QC check was adequate, all other fields were automatically completed as not applicable. Where the QC check score was not interpretable, the subsequent fields of exposure, focus and magnification were provided for the professional photographer to give reasons for the decision.
(XLSX)

Table S2 **Results of the quality assessment conducted by the primary outcome reviewers of the trial.** Results reported for simplicity are the combined quality result, as if either one of the images were suboptimal, the image pair decision was difficult. There were 8 reviewers in the study and quality assessments from all reviewers are included in this table.
(XLSX)

Table S3 **Dataset used to calculate inter-rater reliability and the kappa scores provided.** Reviewers were numbered 5, 6, 7, 9, 10, 12, 14 and 16. When all reviewers scored all image pairs, the number of the reviewers selected for the calculation is listed in columns revA_num and revB_num.
(XLSX)
